# 1298. Validation of Local *Pseudomonas aeruginosa* Risk Factors in Patients with Community-Onset Bacterial Pneumonia

**DOI:** 10.1093/ofid/ofab466.1490

**Published:** 2021-12-04

**Authors:** Morgan Pizzuti, Bailey Smith, Shannon Leighton, Chao Cai, William Lindsey, P Brandon Bookstaver, Joseph Kohn, Majdi N Al-hasan, Hana Winders, Julie Ann Justo

**Affiliations:** 1 Prisma Health Richland - University of South Carolina, Columbia, South Carolina; 2 University of South Carolina College of Pharmacy, Columbia, South Carolina; 3 Prisma Health Midlands, Columbia, South Carolina; 4 University of South Carolina, Columbia, SC

## Abstract

**Background:**

Clinical guidelines for community-acquired pneumonia (CAP) encourage validation of local risk factors for multidrug-resistant organisms. This study aimed to validate previously derived, local risk factors for *Pseudomonas aeruginosa* in patients with community-onset bacterial pneumonia at Prisma Health-Midlands’ hospitals.

**Methods:**

In this retrospective, observational cohort study, adult patients hospitalized with pneumonia MS-DRG codes from January 1, 2017 to March 31, 2020 were randomly screened. Enrolled subjects were admitted to 1 of 3 Prisma Health-Midlands’ hospitals with: diagnosis of pneumonia; receipt of inpatient antibiotics within 48 hours of symptom onset; receipt of over 48 hours of antibiotic therapy; and a causative bacterial pathogen identified via respiratory or blood culture, urinary antigen, or respiratory multiplex PCR panel. Performance of the locally derived score was compared to the Drug Resistance in Pneumonia (DRIP) Score, IDSA 2019 CAP guideline risk factors, and healthcare-associated pneumonia (HCAP) risk factors. Endpoints included sensitivity, specificity, positive and negative predictive value, overall accuracy, and over- and undertreatment rates. Overall accuracy was defined as a case in which the gram-negative antibiotic coverage recommended by the scoring schema would have been appropriate for the identified organism, i.e. neither overtreatment (overly broad-spectrum) nor undertreatment (inadequate spectrum).

**Results:**

Of 713 patients screened, 36 patients met criteria and were enrolled. The most common bacterial pathogens identified were *Pseudomonas aeruginosa* (n = 10, 27.8%) and *Streptococcus pneumoniae* (n = 10, 27.8%). Performance characteristics for each scoring schema are summarized in Table 1.

Table 1. Performance characteristics of risk scores predicting for Pseudomonas aeruginosa community-onset bacterial pneumonia.

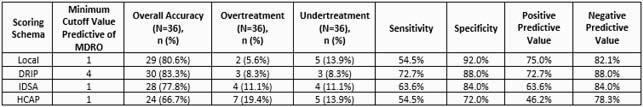

MDRO=multidrug-resistant organism; DRIP=Drug Resistance in Pneumonia Score; IDSA=Infectious Diseases Society of America 2019 Community-Acquired Pneumonia Guideline risk factors ; HCAP=healthcare-associated pneumonia risk factors

**Conclusion:**

Compared to DRIP or IDSA 2019 CAP risk scores, the local risk score performed well at ruling out resistant gram-negatives given its higher specificity and lower overtreatment rate; yet, it did not perform as well at ruling in resistant gram-negatives given a lower sensitivity and undertreatment rate. All scores performed better than HCAP risk factors. Data from this study will be utilized to further refine the local risk score algorithm.

**Disclosures:**

**P. Brandon Bookstaver, Pharm D**, **ALK Abello, Inc.** (Grant/Research Support, Advisor or Review Panel member)**Biomerieux** (Speaker’s Bureau)**Kedrion Biopharma** (Grant/Research Support, Advisor or Review Panel member) **Hana Winders, PharmD, BCIDP**, **biomerieux** (Grant/Research Support) **Julie Ann Justo, PharmD, MS, BCPS-AQ ID**, **bioMerieux** (Speaker’s Bureau)**Merck & Co.** (Advisor or Review Panel member)**Therapeutic Research Center** (Speaker’s Bureau)**Vaxart** (Shareholder)

